# Comparative Phytotoxicity of Leachates from Aircraft and Automobile Tire Wear Particles on Mung Bean (*Vigna radiata* L.) Seed Germination and Seedling Growth

**DOI:** 10.3390/toxics14070587

**Published:** 2026-07-02

**Authors:** Jie Xu, Ning Li, Bingshen Liu, Ying Pan, Yuxin Tian, Yichun Wu, Jian Li, Jianxu Wang, Wenjie Jiang, Tao Wu

**Affiliations:** 1 Department of Bioengineering, Binzhou Polytechnic University, Binzhou 256603, China; xujie@bzpt.edu.cn (J.X.); panying111@163.com (Y.P.); wuyichun05@163.com (Y.W.); 2 Shandong Key Laboratory of Eco-Environmental Science for the Yellow River Delta, Shandong University of Aeronautics, Binzhou 256600, China; lining@stu.sdua.edu.cn (N.L.); liubingshen16@163.com (B.L.); tianyuxin@stu.sdua.edu.cn (Y.T.); 3 College of Biological and Pharmaceutical Engineering, Shandong University of Aeronautics, Binzhou 256600, China; lijiansdua@sdua.edu.cn (J.L.); xusd0509@163.com (J.W.); jiangwenjie@stu.sdua.edu.cn (W.J.)

**Keywords:** tire wear particles, mung bean, leachable additives, phytotoxicity, oxidative damage, environmental risk assessment

## Abstract

Tire wear particles (TWPs) are a significant source of microplastics and chemical additives in the environment; however, differences in the toxicity of particles from different vehicle types remain unclear. This hydroponic study compared the phytotoxicity of leachates from aircraft- and automobile-derived TWPs on mung bean. Both leachates inhibited seed germination and seedling growth, with aircraft TWP leachates showing stronger effects, including greater germination delays and more pronounced reductions in shoot height, root length, and root surface area. Physiological analyses revealed that TWP leachates induced oxidative stress, characterized by significant suppression of superoxide dismutase (SOD) activity, compensatory increases in catalase (CAT) and peroxidase (POD) activities, and marked accumulation of malondialdehyde (MDA), indicating severe membrane lipid peroxidation. Chlorophyll content decreased in all groups, with greater reductions under aircraft leachates. Toxicological Priority Index (ToxPi) modeling identified zinc as the shared primary risk factor, while aircraft tire-specific additives (e.g., dicyclohexylamine, 1,2-dihydro-2,2,4-trimethylquinoline) constituted a distinct risk component linked to differentiated formulations. Aircraft TWP leachates thus exhibit stronger phytotoxicity through multiple pathways. These findings support refined environmental risk assessment and targeted control measures for aircraft TWPs.

## 1. Introduction

Tire wear particles (TWPs) are primarily generated through physical friction between tires and road surfaces, as well as through environmental aging processes such as ultraviolet radiation, mechanical abrasion, and biodegradation [1]. Existing research reveals substantial regional variation in per capita annual emissions of automotive TWPs, ranging from 0.23 to 4.70 kg/year, with a global average of approximately 0.81 kg/year [2]. Most TWPs initially accumulate on road surfaces or roadside areas before being transported via road runoff, stormwater discharges, and wastewater treatment plant effluents to roadside soils or aquatic environments [3]. Once released into the environment, these TWPs are not inert. During the natural aging process, various chemical additives contained within them—such as zinc (Zn), polycyclic aromatic hydrocarbons, and benzothiazoles—are leached into the surrounding environment, generating leachates with potential ecotoxicity [4,5]. Currently, most relevant research focuses on TWPs generated by road transportation [6,7,8], while overlooking this significant emission source in the aviation sector. In real-world environments, the actual levels of TWP in the soil range from 0.007% to 15.8% [9]. Importantly, TWPs can enter farmland through atmospheric deposition, surface runoff, irrigation, and the application of sludge to agricultural land [10,11,12]. Aircraft tires, engineered to withstand the high-speed, high-load conditions of takeoff and landing, often incorporate specialized additives such as carbon-based nanofillers and high-dose antioxidants into their formulations [13,14]. This unique composition, combined with the severe wear conditions experienced during takeoff and landing, may result in heightened ecotoxicity of both TWPs and their leachates. Therefore, systematically elucidating the ecological risks and toxic mechanisms of aircraft tire leachates represents a critical research gap that urgently needs to be addressed.

To ensure tire stability and durability, approximately 5–10% additives are typically incorporated during tire manufacturing [15]. Given the more stringent performance requirements for aircraft tires, the variety and concentration of additives used may be even higher. These additives inevitably leach into aquatic environments, causing harmful effects on aquatic organisms [8]. Previous studies have demonstrated that leachates from TWPs exhibit significant toxicity to aquatic organisms: it not only disrupts the oxidative stress defense system in zebrafish but also causes mortality in copepods and oligochaetes upon high-concentration exposure [16,17,18]. In addition to the parent compounds of additives, their transformation products in the environment also warrant attention. For instance, 6-PPD-quinone, the oxidation product of N-(1,3-dimethylbutyl)-N′-phenyl-p-phenylenediamine (6-PPD), has been identified as a key toxicant responsible for acute salmon mortality in urban rivers [19]. However, existing research has primarily focused on aquatic ecosystems, leaving gaps in understanding the toxicity of TWP leachates in terrestrial environments, particularly their impact on crops. Leachates can enter agricultural soils through irrigation or surface runoff and subsequently be absorbed by plant roots, triggering a series of physiological toxic responses. Therefore, evaluating the toxic effects of TWP leachates on representative crop species and elucidating their stress mechanisms at the physiological and molecular levels is of great scientific importance for comprehensively understanding the terrestrial ecological risks posed by TWPs.

This study selected mung bean (*Vigna radiata* L.) as the test plant, primarily due to its short growth cycle and high sensitivity to environmental pollutant stress [20], which facilitates efficient assessment of toxic effects. Additionally, as a major leguminous crop in Asia [21], research on mung beans holds direct relevance for food safety. Mung beans have been widely employed in ecotoxicological studies to evaluate the impacts of various environmental pollutants [22,23,24]. Building upon this, the present study systematically compared the differential effects of particulate matter leachates from two traffic-related sources—aircraft and automobile TWPs—on mung bean seedling germination, growth, and key physiological and biochemical processes. This was achieved through hydroponic experiments exposing mung bean seedlings to leachates from different brands of aircraft and automobile TWPs. This study employed a multi-tiered assessment framework integrating phenotypic, physiological, biochemical, and chemical risk screening methods to compare the phytotoxicity of leachates from aircraft and automotive tire waste. Unlike previous studies, which primarily focused on tire waste from road transportation and single toxicity indicators, our work incorporates aviation tire waste—a source that has not been fully studied but may represent a significant pollution source. By using the ToxPi model to integrate the chemical composition of leachate with toxicological data, we prioritize the identification of substances responsible for toxicity, thereby enabling a precise understanding of the mechanisms of action of these pollution sources. The specific objectives of this research are as follows: (1) to systematically compare the toxicity of TWP leachates from aircraft and automobiles on mung bean germination and growth; (2) to elucidate the underlying toxicity pathways at the physiological and biochemical levels by measuring key indicators such as chlorophyll content, root and stem growth status, and antioxidant enzyme system activity; and (3) to simulate the potential stress imposed on crops when TWP leachates enter agricultural water bodies, thereby providing experimental evidence for assessing the agroecological risks of microplastics derived from different transportation sources.

## 2. Materials and Methods

### 2.1. Preparation of TWPs and Leachates

To simulate the real-world exposure scenario of TWPs, this study procured two used aircraft tires and two used automobile tires from different companies in Qingdao and Hangzhou, China, respectively, as research subjects (Table 1). According to previous research [6], the tire samples were mechanically abraded using a metal file to generate TWPs. The collected particles were then sieved through a 0.5 mm mesh sieve and stored in the dark. The 0.5 mm mesh size was selected based on previously reported TWP particle size distribution characteristics [6,25]. Particles larger than 0.5 mm were excluded to focus on the size fraction most relevant to environmental transport and bioavailability.

During the leachate preparation stage, the obtained TWPs were mixed with deionized water at a mass concentration of 5% in a 500 mL glass container. The mixture was continuously agitated at 180 rpm at room temperature for 30 days to simulate natural leaching processes, and then filtered through a 0.45 μm membrane filter. The resulting filtrate was used as the TWP leachate stock solution and stored in clean glass containers for further use. However, it should be noted that the soluble chemical components in the leachate could theoretically interact with the filter paper matrix; however, given that filter paper is not designed as an adsorbent and the contact time is limited, such adsorption is generally negligible for most organic additives and metal ions within the concentration range used in this study.

### 2.2. Experimental Plants

Mung bean seeds were sourced from Shouhe Seed Industry Co., Ltd. in Weifang, Shandong Province, China. Prior to the experiment, seeds were selected for plumpness and uniform size. They were disinfected by being soaked in a 3% H_2_O_2_ solution for 30 min, and then rinsed five times with deionized water to completely remove residual disinfectant.

### 2.3. Seed Germination Experiment

Seeds were placed in a Petri dish lined with two layers of moist filter paper and incubated in the dark at 25 °C for 24 h to promote seed germination. The experiment consisted of five treatment groups: one blank control group and four TWP filtrate treatment groups, each with three replicates. Seeds with similar pre-germination radicle lengths were selected. Twenty seeds were evenly distributed in each filter-paper-lined Petri dish, and 10 mL of the corresponding treatment solution was added (distilled water for the control group; 5% TWP filtrate for the treatment groups). The dosage of TWP filtrate employed in this study was determined based on prior research [26]. After sealing, the Petri dishes were placed in a light incubator under the following conditions: day/night temperature of 25 °C, a 12 h/12 h photoperiod, and light intensity of 4000 lx.

### 2.4. Hydroponic Experiment

Hydroponic culture was employed in this study as a standardized screening tool to evaluate the intrinsic phytotoxicity of TWP leachates under controlled conditions, minimizing confounding effects from soil matrix adsorption, organic matter complexation, and microbial degradation. While this approach does not fully represent the complexity of field soil–plant systems, it is widely adopted in plant toxicology for mechanistic investigations and provides essential baseline data for subsequent environmental risk assessments. Seeds were pre-germinated on moist filter paper for 24 h at 25 °C in the dark. Following this, pre-germinated seeds with emerged radicles were immediately transferred to hydroponic containers containing the corresponding treatment solutions (5% TWP leachates or deionized water for the control) and cultured for 7 days under controlled conditions (25 °C, 12 h light/12 h dark photoperiod). During this 7-day period, seedlings developed from germinated seeds to the two-true-leaf stage in the presence of the respective treatment solutions. Thus, pollutant exposure commenced immediately after the 24 h germination period and continued throughout the entire seedling establishment phase. The seedlings were assigned to five experimental groups, consisting of one blank control (BC) group and four treatment groups exposed to 5% leachates derived from AT, AG, CC, and CM tires, respectively. Each container held nine seedlings and was supplied with 100 mL of the corresponding leachate solution. Each experimental group had three replicates. Hydroponic culture was conducted in a climate-controlled incubator under a 12 h light/12 h dark photoperiod at a constant day/night temperature of 25 °C for 7 days. Deionized water was added daily to compensate for evaporative losses and maintain a constant solution volume. All growth and physiological parameters were measured at the end of the cultivation period.

### 2.5. Measurement of Plant Morphological Traits

Shoot length was defined as the vertical distance from the base of the stem to the apex of the stem [27]. Root length was measured using a steel ruler with a precision of 0.1 mm. Seedlings were gently washed and carefully dried with absorbent paper. The root surface area and the number of root tips were quantitatively analyzed based on the images captured with a ScanMaker i800 (Microtek, Shanghai, China). Fresh weight was measured to evaluate the immediate water status and osmotic balance of seedlings under stress, as it reflects cell turgor and membrane integrity. Dry weight, obtained after oven-drying at 105 °C for 30 min followed by 75 °C to constant mass (approximately 48 h), represents the net accumulation of structural biomass (photosynthetic products). The concurrent measurement of fresh and dry weights thus allows assessment of both water relations and true growth, providing complementary information on plant physiological responses to TWP leachate exposure.

### 2.6. Determination of Chlorophyll Content and Seedling Biomass

Chlorophyll content was estimated non-destructively using a Soil and Plant Analyzer Development (SPAD) chlorophyll meter (SPAD-502Plus, Konica Minolta, Tokyo, Japan). Measurements were taken from the most recently fully expanded true leaf of each selected seedling. To eliminate the influence of major veins, SPAD values were recorded at three points (upper, middle, and lower sections) along one side of the leaf blade, meticulously avoiding the midrib and other prominent veins. For each treatment group, five seedlings were randomly selected, and one leaf per seedling was measured following the described procedure. The residual surface filtrate was gently rinsed off with deionized water, and the plants were blot-dried using filter paper. The root system was then separated from the shoots. Fresh weights of the roots and shoots were immediately recorded using a precision analytical balance (PR224ZH/E, OHAUS, Parsippany, NJ, USA) [28]. Subsequently, samples were placed into pre-dried and pre-weighed aluminum boxes and oven-dried. An initial deactivation period of 30 min at 105 °C was followed by drying at 75 °C until a constant weight was achieved (approximately 48 h). After drying, the samples were cooled in a desiccator and weighed to determine root and shoot dry weights.

### 2.7. Biochemical Analysis

To evaluate the oxidative damage induced in mung beans following exposure to TWP leachates, this study assessed changes in key oxidative stress biomarkers. Upon completion of the exposure experiment, mung bean samples from the BC (blank control) and the 5% TWP leachate exposure groups were selected for biochemical analysis. The activity levels of superoxide dismutase (SOD), catalase (CAT), peroxidase (POD), and malondialdehyde (MDA) were determined using commercial kits from Sangon Biotech (Shanghai, China) Co., Ltd. All assays were performed strictly according to the kit protocols, with three replicates conducted to ensure data reliability.

Toxicological Priority Index (ToxPi) integrates multi-source data—including chemical hazard information, safety profiles, and exposure data—into a transparent, visual ranking system, enabling the comprehensive presentation of multi-dimensional evidence [29]. By fusing diverse datasets, this tool synthesizes multifaceted evidence, such as compound hazard severity, exposure potential, and biological activity, into an intuitive, unified ranking, thereby providing essential support for prioritizing environmental risk management [30].

To assess the potential risks posed by organic compounds in four types of TWP leachates, this study employed ultrahigh-performance liquid chromatography-tandem 4500 triple quadrupole mass spectrometer (UPLC-MS/MS; Applied Biosystems, Framingham, MA, USA) to determine the concentrations of organic compounds. Specific parameters are provided in Text S1. Twenty-five target organic compounds were selected from the analysis. These compounds, together with seven simultaneously detected heavy metal elements, were subjected to priority assessment using the ToxPi model (version 2.3). For heavy metal analysis, the seven target elements in the TWP leachates were quantified using inductively coupled plasma mass spectrometry (ICP-MS, Agilent 7900, Agilent Technologies, Santa Clara, CA, USA). Calibration standards were prepared from certified multi-element stock solutions (Agilent, Santa Clara, CA, USA), and internal standards (In, Rh, Re) were added online to correct for matrix effects and signal drift. All samples were analyzed in triplicate, and quality control was assured by running certified reference materials and procedural blanks.

### 2.8. Statistical Analysis

All data are presented as the mean ± standard deviation (SD). Data preprocessing was performed using Microsoft Excel 2021. Statistical analyses were conducted using SPSS 27.0 (IBM Corp., Armonk, NY, USA), with one-way analysis of variance (ANOVA) followed by Tukey’s post hoc test. A *p*-value of < 0.05 was considered statistically significant. Groups sharing the same letter are not significantly different from each other at *p* < 0.05, while groups with different letters indicate statistically significant differences (*p* < 0.05). Data visualization was performed using Origin 2021 software.

## 3. Results and Discussion

### 3.1. Germination Response of Mung Beans to Different TWP Leachates

**Figure 1:**
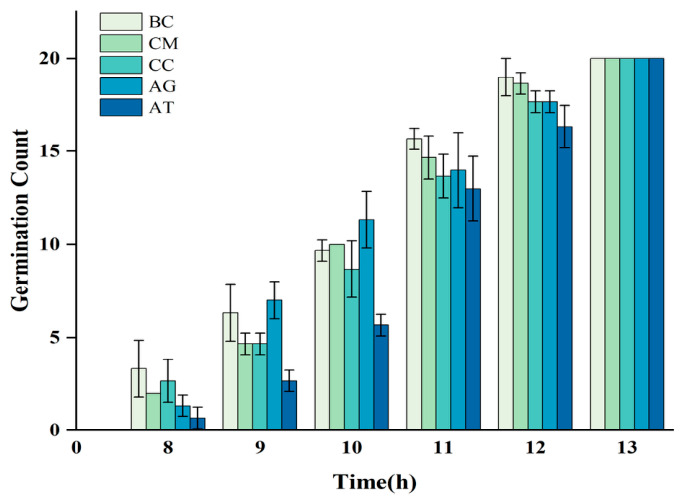
Effect of leachates from TWPs of automobile tires (CM, CC) and aircraft tires (AG, AT) on mung bean seed germination. Values are expressed as the mean ± standard deviation of three replicates for each treatment.

As shown in Figure 1, although all leachate treatments ultimately achieved a final germination rate of 100%, comparable to that of the BC, they significantly delayed the germination process. Specifically, at the early stage (8 h), the germination count in the 5% aircraft tire leachate groups (AT, AG) was significantly lower than that in the BC. A similar but less pronounced inhibitory trend was also observed in the 5% automobile TWP leachate-exposed groups (CM, CC). This indicates that chemical additives in TWP leachates may have disrupted early metabolic activities during seed germination, such as water absorption and respiration [31]. Although radicle penetration through the seed coat was not completely inhibited, the germination process was delayed, indirectly confirming the phytotoxicity of these leachates. This delayed germination may be attributed to the combined toxic effects of organic additives and heavy metal ions present in the leachate [32]. On one hand, heavy metals such as Zn^2+^ can impose stress on seeds during the initial germination stage. Previous studies have shown that high concentrations of Zn^2+^ disrupt water balance and compromise the integrity of cell membranes in plants, while suppressing the activity of key hydrolases, thereby delaying seed swelling and the initiation of metabolic activity [33]. On the other hand, multiple organic additives used in tires have been shown to exhibit phytotoxicity, potentially inhibiting seed respiration through disruption of energy metabolism [34]. In this study, aircraft TWP leachates exhibited stronger inhibitory effects than automobile TWP leachates. This may be attributed to the use of different types or higher doses of chemical additives required to meet more stringent performance standards (e.g., heat resistance and shear resistance), resulting in higher concentrations of toxic substances or more biotoxic components in the leachate.

### 3.2. Morphological Alterations in Seedlings Under Leachate Exposure

**Figure 2:**
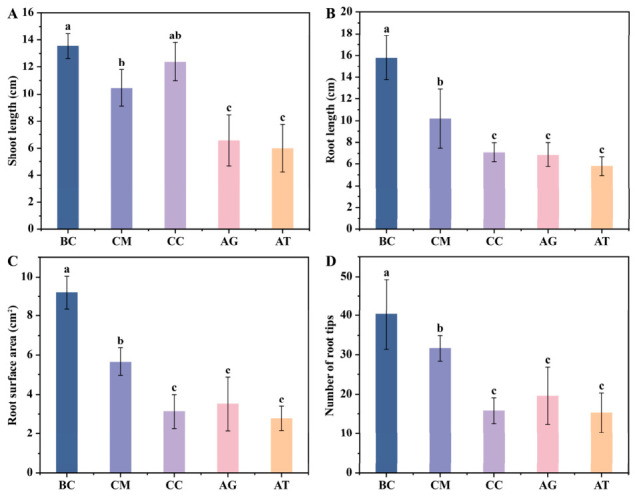
Effect of leachates from TWPs of automobile tires (CM, CC) and aircraft tires (AG, AT) on the growth morphology of mung bean seedlings. (**A**), shoot length; (**B**), root length; (**C**), root surface area; (**D**), number of root tips. Values are expressed as the mean ± standard deviation of five replicates for each treatment. Different letters indicate significant differences among treatments (*p* < 0.05, Tukey’s post hoc test).

To further evaluate the phytotoxic effects of different TWP leachates, the morphological traits of seedlings were examined. Regarding shoot length, the treatment groups exhibited the following order: BC > CC > CM > AG > AT (Figure 2A). This indicates that aircraft TWP leachates (particularly AT) exerted the strongest inhibitory effect on the above-ground elongation growth of mung beans. This result may be attributed to specific organic additives incorporated into aircraft tires to accommodate higher-intensity operating conditions [10]. Upon leaching, these additives likely cause more pronounced interference with plant cell elongation [35], potentially affecting the metabolism and signaling of endogenous plant hormones, thereby inhibiting stem elongation [36]. The overall trends observed in root length (Figure 2B) and root surface area (Figure 2C) indicated that aircraft TWP leachates generally exerted stronger inhibition on root growth, while automobile TWP leachate exhibited specific effects on different physiological parameters. This difference likely stems from variations in leached components or physical properties, which may originate from distinct tire formulations and particle characteristics that influence plant roots [37]. However, for root tip number—a key indicator reflecting root branching capacity (Figure 2D)—the inhibition pattern differed from that observed in plant height, with treatment groups showing the following order: BC > CM > AG > CC > AT. Notably, the CM group exhibited greater effects on shoot height than the CC group, yet its root tip count was significantly higher than that of the latter. This suggests that the CC group may specifically inhibit lateral root development, implying potential differences in the toxicity mechanisms of TWP leachate from different sources.

The results indicate that the effects of TWP leachates on plant growth are not attributable to a single inhibitory mechanism but rather arise from the combined action of the specific toxicity of released chemicals [38]. This interference forces plants to make trade-offs in resource allocation, triggering adaptive changes in morphogenesis. Such responses may represent a survival strategy for plants under stressful environmental conditions [39].

### 3.3. Effects of TWP Leachates on Biomass Accumulation and Chlorophyll Content

**Figure 3:**
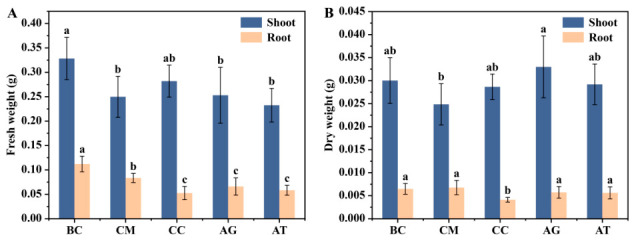
Changes in fresh and dry biomass of mung bean seedlings under exposure to leachates from automobile tire wear particles (CM, CC) and aircraft tire wear particles (AG, AT). (**A**) Fresh biomass of mung bean seedlings; (**B**) dry biomass of mung bean seedlings. All data are shown as mean ± SD (*n* = 5). Different letters indicate significant differences among treatments (*p* < 0.05, Tukey’s post hoc test).

The leachates from TWPs exerted complex effects on biomass accumulation in mung bean seedlings, and significant differences were observed between shoot weight and root weight (Figure 3). In terms of fresh weight, BC maintained the highest values for both shoot and root weight, which further confirms that TWP leachate exerts a stress effect on plant growth (Figure 3A). However, the extent of inhibition differed across treatment groups. The CC treatment demonstrated relatively moderate inhibition on shoot fresh weight; however, its root fresh weight was the lowest among all treatments. This implies that specific components in CC leachate may impose a strong, organ-specific inhibitory effect on root growth or water retention capacity. Regarding the dry weight of plants, the results were more intricate, with certain treatments even exhibiting higher dry weights than the BC group (Figure 3B). Notably, both shoot dry weight in the AG treatment and root dry weight in the CM treatment surpassed those of the BC group. This phenomenon might suggest that the improvement in physiological parameters under specific TWP leachate could be attributed to proactive adaptive physiological regulation initiated by organisms to cope with stress [40]. Similar observations have been reported in studies on environmental pollutant stress [41,42,43], potentially leading to increased dry matter accumulation ratios. The root dry weight of the CC treatment consistently remained the lowest among all groups, further supporting the inference that TWP leachate significantly impairs root growth or physiological function. The patterns of changes in fresh weight and dry weight suggest that the impact of leachates on plant biomass is not a straightforward linear inhibition. It might reduce the plant’s water uptake and retention capacity via toxic effects (manifested as a decrease in fresh weight), or it could serve as an environmental stress signal, modifying the plant’s material allocation and metabolic strategies (reflected in changes in dry weight ratios) [31]. The differentiation between aboveground and underground reaction modes demonstrates the complexity of the ecotoxicology of TWP leachates.

**Figure 4:**
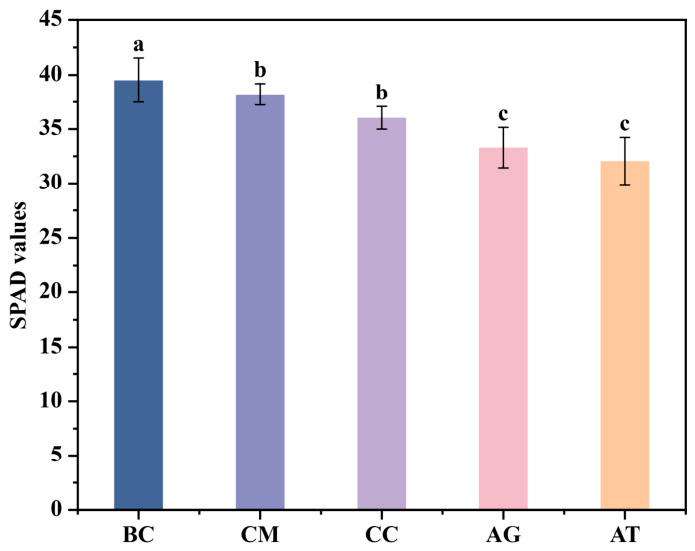
Effect of leachates from TWPs of automobile tires (CM, CC) and aircraft tires (AG, AT) on chlorophyll content (SPAD values) in mung bean leaves. Values are expressed as the mean ± standard deviation (*n* = 5). Different letters indicate significant differences among treatments (*p* < 0.05, Tukey’s post hoc test).

The chlorophyll content in plant leaves serves as a key indicator that reflects their photosynthetic capacity and physiological health [44]. As depicted in Figure 4, the leachate significantly inhibited the SPAD values of chlorophyll in mung bean leaves (*p* < 0.05), and the degree of inhibition was associated with the tire type. All leachate groups exhibited significantly lower chlorophyll content compared to the BC group. Although the automobile TWP leachate-exposed group had a lower value than the control, it was still significantly higher than the aircraft TWP leachate-exposed group, suggesting that TWP leachates from aircraft tires have a stronger inhibitory impact on chlorophyll synthesis. The decline in chlorophyll content is typically associated with the accumulation of reactive oxygen species (ROS) under abiotic stress [45]. To meet more stringent performance standards, aircraft tires may utilize rubber formulations with higher concentrations or more biotoxic chemical additives [9,10]. When these substances seep into leachates, they can be absorbed by plant roots and transported to leaves. There, they may induce intense oxidative stress, directly damaging the molecular structures of chlorophyll or inhibiting the activity of key synthesis enzymes [31]. Furthermore, heavy metals such as Zn^2+^ in the leachate can displace Mg^2+^ from the chlorophyll center when their concentrations surpass plant tolerance thresholds, resulting in chlorophyll inactivation and disruption of the thylakoid membrane structure. These effects jointly lead to a substantial reduction in SPAD values [46]. The decrease in chlorophyll content, particularly in the group treated with aircraft tire leachate, further validates from the perspective of photosynthetic organ physiology that TWP leachates, especially those derived from aircraft tires, impose severe physiological stress on mung bean seedlings. We also acknowledge that the use of SPAD as a chlorophyll indicator, while providing a rapid and non-destructive assessment, does not allow for quantitative differentiation of Chl a, Chl b, and carotenoids. Future investigations would benefit from incorporating full-spectrum spectrophotometric analyses to capture potential shifts in Chl a/b ratios and carotenoid composition, which may offer deeper insights into PSII photoprotective mechanisms.

### 3.4. Oxidative Stress and Activation of the Antioxidant Defense System

**Figure 5:**
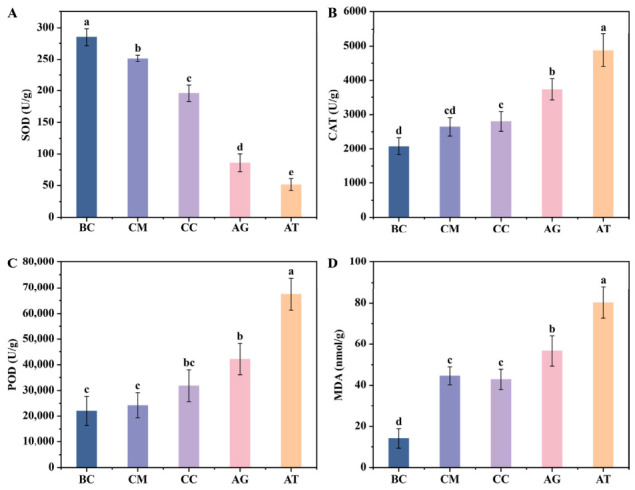
Effect of leachates from TWPs of automobile tires (CM, CC) and aircraft tires (AG, AT) on oxidative stress levels of mung beans. (**A**) SOD; (**B**) CAT; (**C**) POD; (**D**) MDA. Values are expressed as the mean ± standard deviation (*n* = 3). Different letters indicate significant differences among treatments (*p* < 0.05, Tukey’s post hoc test).

When plants respond to external stress, they activate their antioxidant defense systems, which are accompanied by dynamic changes in membrane lipid peroxidation levels [40]. This study demonstrated that TWP leachates derived from different sources exerted significant and systematic impacts on the oxidative stress response of mung bean seedlings (Figure 5). SOD, which serves as the first line of defense against ROS, exhibited significantly inhibited activity under TWP leachate treatment (Figure 5A). The most pronounced suppression was observed in the aircraft TWP leachate-exposed groups (AT, AG). This observed hormetic response is consistent with findings reported in nanoplastic stress studies, where low-level oxidative stress has been shown to activate plant antioxidant defense systems [47]. This result may originate from the direct binding of heavy metals (e.g., Zn^2+^) in the leachate to the active site of the enzyme or the interference of organic additives with the synthesis and function of the enzyme [48]. Notably, while the activity of SOD was suppressed, the activities of CAT and POD—downstream antioxidant enzymes—showed inducible increases in the TWPs-treated groups, particularly in the aircraft TWP leachate-exposed groups (Figure 5B,C). When insufficient SOD activity leads to reduced O_2_^−^ scavenging efficiency, intracellular H_2_O_2_ levels rise accordingly, thereby activating enzyme systems such as CAT and POD, which decompose H_2_O_2_ and prevent its further conversion into the more destructive hydroxyl radical (OH) [26]. However, this study observed a significant increase in MDA content (Figure 5D), with the most severe MDA accumulation occurring in the aircraft TWP leachate-exposed groups. MDA, the end product of membrane lipid peroxidation, directly confirms that ROS have caused substantial damage to the cell membrane system [49]. This suggests that aircraft TWP leachate may have triggered more intense or prolonged ROS bursts, exceeding the clearance capacity of the plant’s endogenous antioxidant system and leading to irreversible accumulation of oxidative damage. These findings disclose disparities in the toxic impacts of the two types of TWP leachates at the oxidative stress level, validating previously observed growth inhibition phenotypes. While the coordinated changes in SOD, CAT, POD, and MDA activities provide evidence of oxidative stress under TWP leachate exposure, these parameters are inherently variable and responsive to multiple stressors. Therefore, our data should be interpreted as evidence of stress onset rather than as a definitive mechanistic pathway. Future studies integrating transcriptomic or proteomic analyses are needed to elucidate the specific molecular mechanisms underlying the observed phytotoxic effects.

### 3.5. Toxicity-Driven Prioritization of Leached Tire Additives

**Figure 6:**
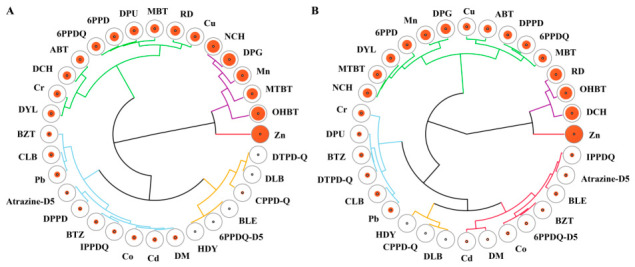
Priority ranking of organic compounds and heavy metals in automobile tire leachates (**A**) and aircraft tire leachates (**B**) based on ToxPi model assessment.

The concentrations of 25 organic compounds and 7 heavy metals in TWP leachates from the four tire brands are shown in Table S1. To systematically identify key risk substances in the leachates, this study employed the ToxPi for a comprehensive assessment of the detected organic pollutants and heavy metals. As shown in Figure 6, the risk substances in automobile and aircraft TWP leachates exhibit both commonalities and significant differences. Heavy metal Zn^2+^ ranked first in the ToxPi analysis for both types of leachates, indicating its primary contribution to the ecological toxicity of TWPs. This finding aligns closely with extensive prior research [17,50,51,52] and traces the observed plant growth inhibition and oxidative stress in this study back to their source. Furthermore, manganese (Mn) ranked among the top six in both lists, which further confirms that heavy metal leaching is one of the core toxic pathways of TWPs. The risk ranking of organic additives also showed distinct differences between the two types of leachates. In automotive tire leachate, substances such as 2-benzothiazolol (OHBT) and 1,3-diphenylguanidine (DPG) ranked among those with the highest concentrations (Figure 6A). These compounds are commonly used as vulcanization accelerators or antioxidants. In contrast, aircraft TWP leachate contains distinct priority substances. Specifically, dicyclohexylamine (DCH) and 1,2-dihydro-2,2,4-trimethylquinoline (RD) ranked significantly higher in the leachate of TWPs compared to their rankings in the leachate of automotive TWPs (Figure 6B). Typically, RD shows high persistence and bioaccumulation capabilities, as it can resist rapid degradation in the environment [53]. This resistance consequently prolongs its contact time with soil organisms. Meanwhile, DCH may display strong biotoxicity and mobility [54]. In conventional automotive tires, both of these compounds are either present at lower levels or completely absent, which underscores the distinctive ecological risks associated with aircraft TWPs. This discrepancy probably originates from the specialized rubber formulations of aircraft tires that are designed for extreme operating conditions, such as higher takeoff and landing speeds and temperatures, which leads to increased incorporation and leaching of specific additives [9,10]. The oxidation product 6PPD-quinone (6-PPDQ) of the tire ozone stabilizer N-(1,3-dimethylbutyl)-N′-phenyl-p-phenylenediamine (6-PPD) was detected in both types of leachates, and its ToxPi scores were within the higher-risk range. 6-PPDQ has attracted substantial attention because of its acute toxicity to aquatic organisms, especially salmonids [55]. The ToxPi ranking results clearly reveal that Zn^2+^ is the primary universal toxicant in TWP leachates. It should be noted, however, that this identification is based on leachate composition analysis and existing toxicological data, rather than direct quantification of zinc accumulation in plant tissues, nor does it include analysis of heavy metal-specific detoxification responses (e.g., glutathione, phytochelatins). As an essential micronutrient, Zn exhibits toxicity only above species-specific threshold concentrations. Future studies should employ tissue elemental analysis combined with targeted measurements of glutathione and phytochelatin levels, as well as the activities of key biosynthetic enzymes such as phytochelatin synthase and glutathione reductase, to confirm the causal role of Zn and to distinguish its contribution from that of organic additives. Meanwhile, the specific organic additives unique to tires from different sources constitute the chemical foundation for their differential risks, offering direct scientific evidence for the targeted management of high-risk tire chemicals.

## 4. Conclusions

This study systematically compared the toxic effects and underlying mechanisms of leachates from aircraft and automobile TWPs on mung bean seedlings. Results indicated that both types of leachates significantly inhibited mung bean seed germination and seedling growth, with aircraft TWP leachates (AT, AG) exhibiting greater toxicity. Physiological and biochemical analyses revealed that the core toxic mechanism involves inducing oxidative stress. Specifically, the leachate of TWPs inhibited the activity of SOD, while it activated CAT and POD as compensatory responses. Nevertheless, this still resulted in a significant increase in MDA levels, leading to irreversible damage from membrane lipid peroxidation. The differences in toxicity were closely associated with the components of the leachate. Zn^2+^ was a prevalent major risk factor, and additives specific to aircraft TWP leachates, such as DCH and RD, further exacerbated ecological risks. Although our hydroponic experiments indicate that TWP leachate exhibits inherent phytotoxicity during the early seedling establishment stage, several limitations must be acknowledged. First, due to the lack of an isotonic control group, it is not possible to clearly distinguish between the effects of chemical toxicity and osmotic effects; however, the priority substances identified by ToxPi and the differential responses among different leachate types both suggest that chemical toxicity is the primary driving factor. Second, due to substrate interactions and developmental regulation, plant responses may vary depending on soil conditions or different developmental stages. Third, although it is known that tire leachate alters the pH and electrical conductivity of the exposure medium and may induce nonspecific stress, these parameters were not monitored in this study. Future research should incorporate isotonic control groups, soil-based systems, multiple developmental stages, and real-time monitoring of pH and electrical conductivity to strengthen causal inferences and validate their environmental relevance. This study verified that aircraft TWPs demonstrated more pronounced phytotoxicity, which provided a scientific foundation for differentiated environmental risk assessment and precise management of traffic-derived microplastics. Future research should focus on (1) the long-term impacts of TWP leachates in realistic soil–plant systems; (2) the individual and synergistic toxicity mechanisms of key characteristic pollutants (e.g., specific organic additives) in the leachates; (3) establishing a toxicity-based source identification method for TWPs to support the development of environmental standards.

## Figures and Tables

**Table 1:** Specific parameters of aircraft tires and automobile tires from different brands used in the preparation of TWPs.

Brand	Abbreviation	Sidewall Markings	Suitable for the Vehicle Models	Manufacturing Date
THREE Ring	AT	40 × 16–16	Airplane	March 2021
Goodyear	AG	46 × 17–20	Airplane	July 2022
CHAO YANG	CC	205/55 R16	Sedan	May 2021
MICHELIN	CM	205/55 R16	Sedan	May 2020

## Data Availability

Data are included in this article or Supplementary Materials.

## Supplementary Materials

The following supporting information can be downloaded at: https://www.mdpi.com/article/10.3390/toxics14070587/s1. Table S1: The concentrations of 25 organic compounds and 7 heavy metals in four types of tire leachate (µg kg^−1^); Text S1: UPLC-MS/MS analytical conditions.
