# Supplementary material for: Comparative Phytotoxicity of Leachates from Aircraft and Automobile Tire Wear Particles on Mung Bean (Vigna radiata L.) Seed Germination and Seedling Growth
Source: Toxics. 2026 Jul 2;14(7):587. doi: 10.3390/toxics14070587 (PMC13417233; doi:10.3390/toxics14070587)
Supplement: Supplementary file 1 [file toxics-14-00587-s001.zip › toxics-4387690-supplementary.pdf]

Text S1. UPLC-MS/MS analytical conditions.

All target analytes (TAs) in different samples were quantified using an EXion AD ultrahigh performance liquid chromatography-tandem 4500 triple quadrupole mass spectrometer (UPLC-MS/MS, Applied Biosystems, Framingham, MA). A BEH C18 column (100 × 2.1 mm, 1.7 μm; Waters) connected in series to an EVO C18 guard column (5 × 2.1 mm, 1.7 μm; Phenomenex) was employed for chromatographic separation. The column temperature was set at 35 oC. Ultrapure water containing 0.001% formic acid (A) and methanol (B) was used as the mobile phase. The sample injection volume was 10.0 μL, and the flow rate was 0.3 mL/min. The gradient of the mobile phase was programmed as follows: 0-1.0 min, 5% B; 1.0-3.5 min, 5% B-60% B; 3.5-8.0 min, 60% B-95% B; 8.0-10.0 min, 95% B; 10.0-10.1 min, 95% B-5% B; and 10.1-12.0 min, 5% B. An electrospray ionization (ESI) source was selected for simultaneous scanning detection in positive (ESI+) and negative (ESI-) ion modes. The ion spray voltage was 5500 V for ESI+ and -4500 V for ESI-.

Table S1. The concentrations of 25 organic compounds and 7 heavy metals in four types of tire leachate (μg kg^{-1})

items	Brand			
	AG	AT	CC	CM
Cd	0.07	0.06	0.03	0
Cr	1.33	0.79	0.67	2.22
Co	0.17	0.08	0.07	0.57
Cu	2.31	3.75	7.07	8.22
Mn	46.64	24.09	23.16	84.49
Pb	0.65	0.31	0.3	0.47
Zn	13991	11167	3335.1	987.98
2-Aminobenzothiazole (ABT)	21.98	0.79	2.22	2.48
2-Benzothiazolol (OHBT)	239.87	222.98	245.54	146.07
2-Methylthio-1,3-Benzothiazole (MTBT)	9.47	16.66	7.18	71.41
1,3-Diphenylguanidine (DPG)	6.02	148.46	46.03	114.16
N-(1,3-dimethylbutyl)-N-phenyl-p-phenylenediamine (6PPD)	85.93	4.31	3.34	7.03
N-Cyclohexylformamide (NCH)	19.84	8.13	47.23	119.65
Dicyclohexylamine (DCH)	270.54	905.19	13.92	0.47
6PPD-Quinone (6PPDQ)	10.62	5.05	4.45	4.59
9,9-dimethylcarbazine(BLE)	0.07	0.57	0.01	0.01
N,N-Diethyl-1,4-phenylenediamine (DPPD)	11.26	4.73	0.46	0.03
N,N-Dicyclohexylmethylamine (DYL)	2.32	58.52	1.13	0
N,N'-Diphenylurea (DPU)	0.1	0.84	1.47	18.87
6PPDQ-D5	0.01	0	0	0
Atrazine-D5	0	0.02	0.01	0
2,2'-Dithiobis(benzothiazole) (DM)	0.02	0.11	0.02	1.45
1,2-dihydro-2,2,4-trimethylquinoline (RD)	71.41	312.28	8.53	9.85
2,5-Cyclohexadiene-1,4-dione, 2-[(1-methylethyl)amino]-5-(phenylamino) (IPPDQ)	0.01	2.5	0.04	0.5
2,5-Cyclohexadiene-1,4-dione, 2-(cyclohexylamino)-5-(phenylamino) (CPPD-Q)	0.01	0.01	0.04	0.06
N,N'-Bis(methylphenyl)-1,4-benzenediamine (DTPD-Q)	2.37	0.04	0.03	0.02
Benzotriazole (BTZ)	0.29	0.34	0.13	0.18
1-Hydroxybenzotriazole (HDY)	0.04	0.01	0	0
5-Chlorobenzotriazole (CLB)	0.56	0.5	0.55	0.31
5,6-Dimethylazimidobenzene (DLB)	0	0	0.03	0.02
2-Mercaptobenzothiazole (MBT)	2.28	15.89	0.75	22.26
5-Methyl-1H-benzotriazole (BZT)	0.03	0.34	0	0.07
